# Supplementary material for: i-shaped antibody engineering enables conformational tuning of biotherapeutic receptor agonists
Source: Nat Commun. 2024 Jan 20;15:642. doi: 10.1038/s41467-024-44985-x (PMC10799922; doi:10.1038/s41467-024-44985-x)
Supplement: Supplementary file 1 — Supplementary Information [file 41467_2024_44985_MOESM1_ESM.pdf]

Supplementary Information

i-shaped antibody (iAb) engineering enables conformational tuning of biotherapeutic receptor agonists

Matthew G. Romei[1^], Brandon Leonard[1^], Zachary B. Katz[2], Daniel Le[3], Yanli Yang[1], Eric S. Day[4], Christopher W. Koo[5], Preeti Sharma[1], Jack Bevers III[1], Ingrid Kim[1], Huiguang Dai[1], Farzam Farahi[1], May Lin[6], Andrey S. Shaw[2], Gerald Nakamura[1], Jonathan T. Sockolosky[1]*, Greg A. Lazar[1]*

[1]Department of Antibody Engineering, Genentech Inc., South San Francisco, CA, USA
[2]Department of Research Biology, Genentech Inc., South San Francisco, CA, USA
[3]Department of Microchemistry, Proteomic, Lipidomics, and Next Generation Sequencing, Genentech Inc., South San Francisco, CA, USA
[4]Department of Research Biology, Genentech Inc., South San Francisco, CA, USA
[5]Department of Structural Biology, Genentech Inc., South San Francisco, CA, USA
[6]Department of Pharma Technical Development, Genentech Inc., South San Francisco, CA, USA
^These authors contributed equally to this work
*Correspondence: Greg A. Lazar, gregorylazar@gmail.com; Jonathan T. Sockolosky, jsockolo@gmail.com

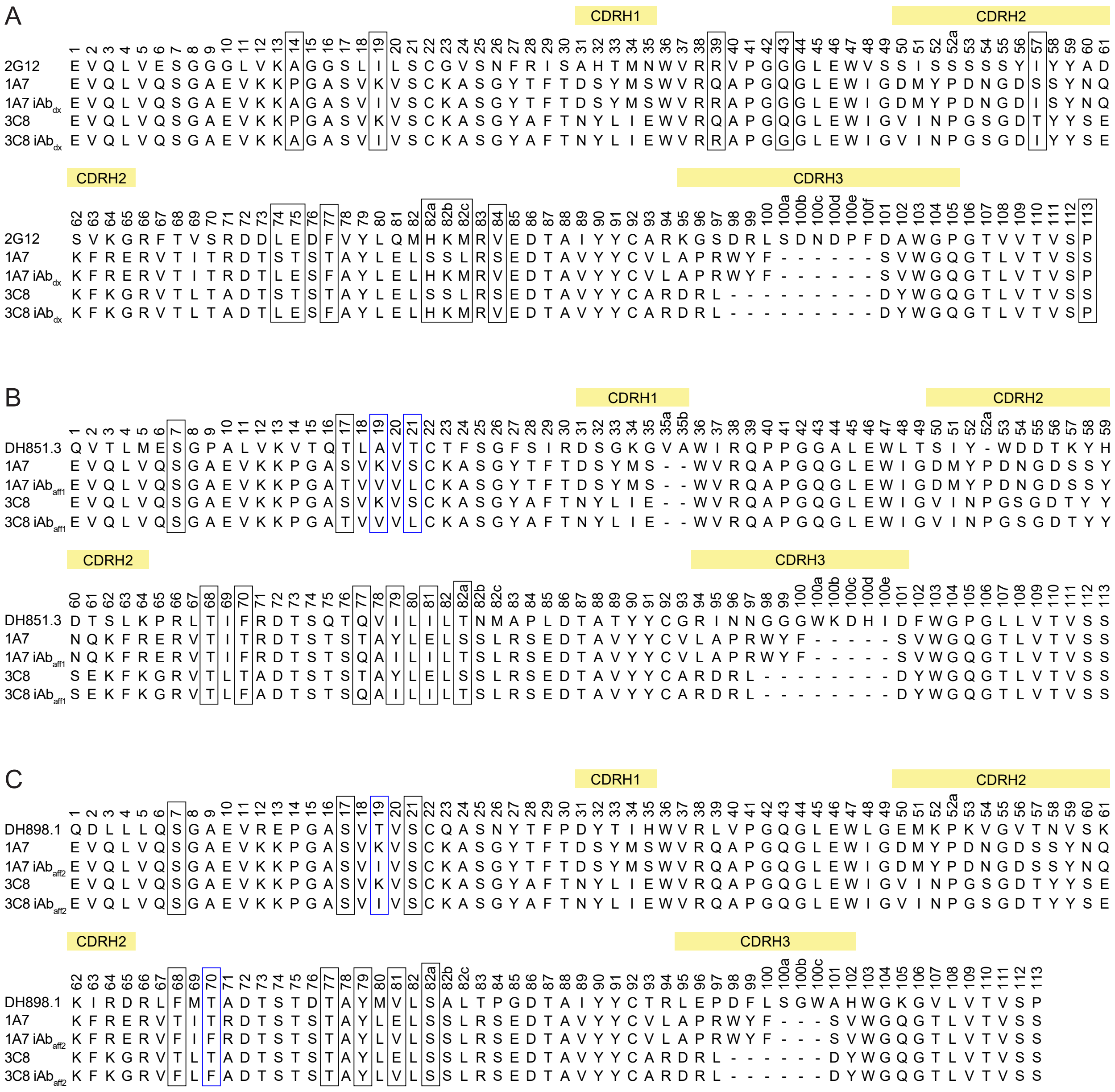

Supplementary Figure 1. Select examples of iAb engineering. Sequence alignments of previously described iAbs [2G12 (A), DH851.3 (B), or DH898.1 (C)], WT anti-OX40 clones (1A7 and 3C8), and their respective iAb mutation set engrafted formats. Black boxed residues show positions where the anti-OX40 clone sequence was changed to that of the indicated iAb in order to engineer iAb formation. Blue boxes indicate additional, non-native hydrophobic substitutions previously shown to strengthen the affinity interfaces from the DH851 and DH898 lineages. In some cases, the boxed residue was the same between the original iAb and the anti-OX40 antibody, and no sequence alteration was needed.

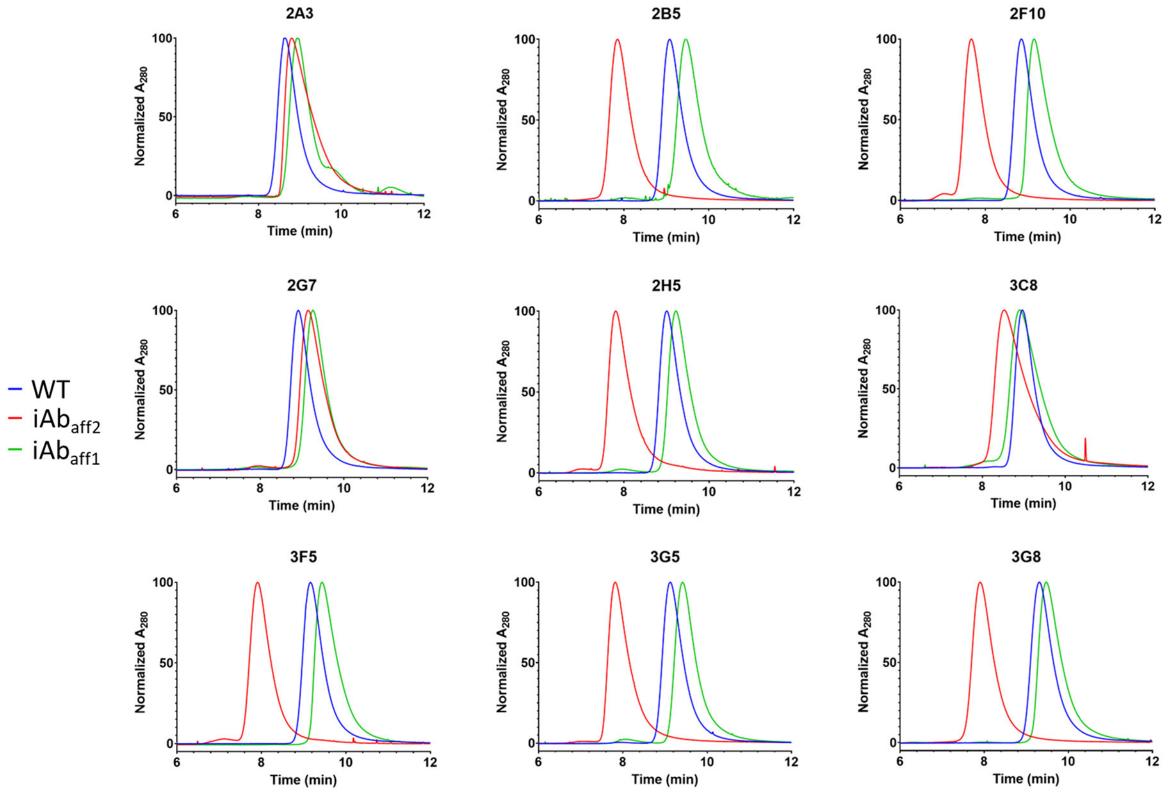

Supplementary Figure 2. Analytical size exclusion chromatography analysis of WT and iAb anti-OX40 antibody clones. The iAb_{aff1} clones are monomeric similar to WT, with a general trend of a shift toward longer elution times for the iAb. Small differences in elution time can be attributed to either the difference in shape (i- vs Y-shaped antibodies) and/or differences in column interaction from the introduction of additional hydrophobic residues. 7 of the 9 iAb_{aff2} clones elute at earlier times more consistent with antibody dimers. The dimerization of iAb_{aff2} is explored further in Fig. S3.

A

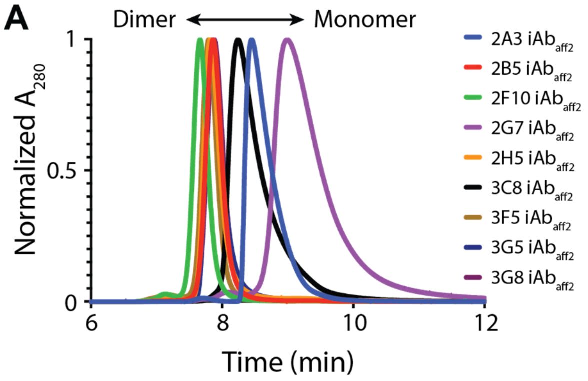

B

Sample	SEC-MALS Molecular Weight (Da)	Species
2A3 iAb_{aff2}	152,200	Monomer
2B5 iAb_{aff2}	284,300	Dimer
2F10 iAb_{aff2}	289,600	Dimer
2G7 iAb_{aff2}	163,600	Monomer
2H5 iAb_{aff2}	250,500	Dimer
3C8 iAb_{aff2}	226,800	Mixture
3F5 iAb_{aff2}	264,000	Dimer
3G5 iAb_{aff2}	277,400	Dimer
3G8 iAb_{aff2}	291,800	Dimer

C

Sample	Concentration (mg/ml)	SEC-MALS Molecular Weight (Da)
3C8 iAb_{aff2}	4.9	243,600
3C8 iAb_{aff2}	1.9	225,200
3C8 iAb_{aff2}	0.38	204,100

Supplementary Figure 3. Solution behavior of iAb_{aff2} anti-OX40 antibody clones. A) Analytical SEC chromatograms of the iAb_{aff2} anti-OX40 clones show a range of elution times. B) Table of SEC-MALS data quantitatively characterizes each iAb_{aff2} clone as a monomer, dimer, or mixture of the two. C) Concentration dependence of SEC-MALS molecular weight for iAb_{aff2} 3C8. As the sample is diluted, the sample becomes more monomeric, demonstrating that the iAb interaction is in equilibrium.

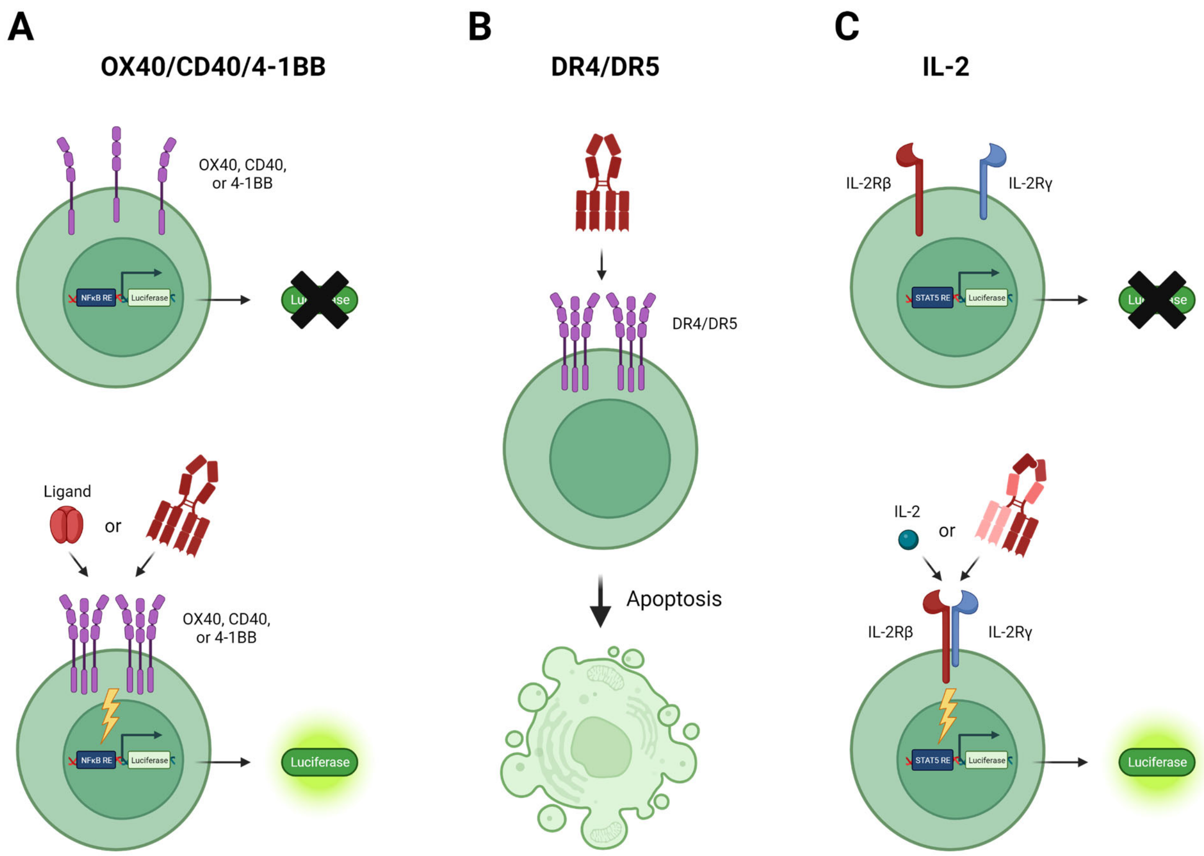

Supplementary Figure 4. Schematic depicting cell-based reporter assays. (A) OX40, CD40, and 4-1BB receptor agonism was determined using an NFκB-luciferase reporter cell line engineered to over-express the desired receptor. Agonism of these receptors drives downstream NFκB signaling, which is assessed through the expression of luciferase. (B) DR4 and DR5 receptor agonism was determined using Colo-205 cells that endogenously express both receptors. Agonism of either receptor results in cell death, which was measured using CellTiter-Glo. (C) Agonism of the IL-2 pathway was determined using a Jurkat-based STAT5-luciferase reporter cell line that was engineered to over-express IL-2Rβ and IL-2Rγ. Heterodimerization of IL-2Rβ and IL-2Rγ results in downstream STAT5 signaling, which is assessed through the expression of luciferase. The schematics were created using BioRender.com. See Methods section for more details on each assay.

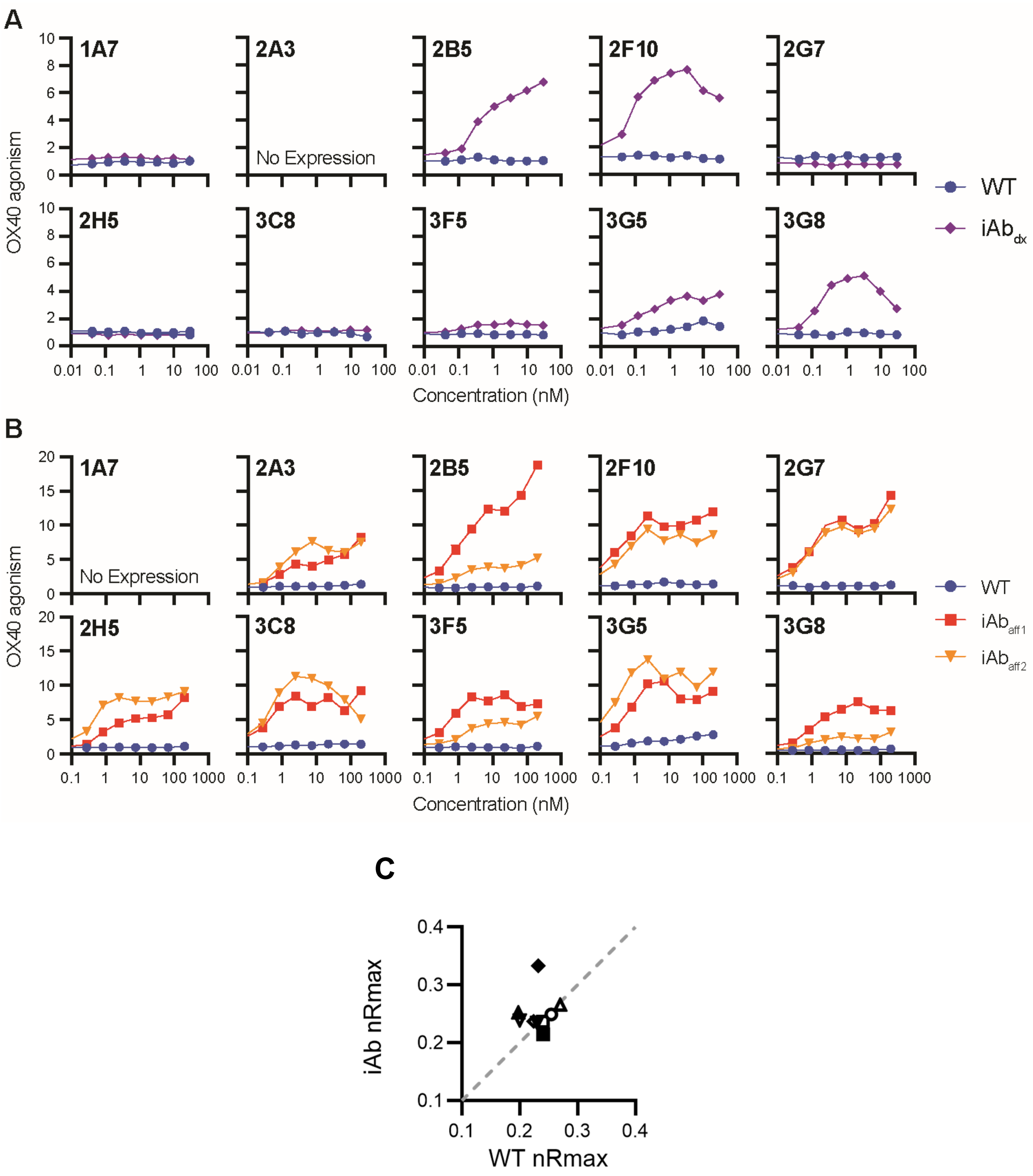

Supplementary Figure 5. OX40 agonism activity and binding of iAb inducing mutation sets. A and B) Individual titrations of 10 anti-OX40 clones engrafted with either the iAb_{dx} (A) or affinity-based (B) iAb inducing mutation sets. The data are shown as fold change over an untreated control (n=2 independent wells). 2A3 did not express with the iAb_{dx} mutation set, while 1A7 did not express with either affinity-based mutation set. C) Surface plasmon resonance (SPR) data comparing normalized Rmax (nRmax) values for each anti-OX40 clone as either an iAb_{aff1} or WT IgG. The dotted gray line has a slope of 1 and indicates no change between the two formats. See Figure 2B for the relevant legend for (C). Source data are provided as a Source Data file.

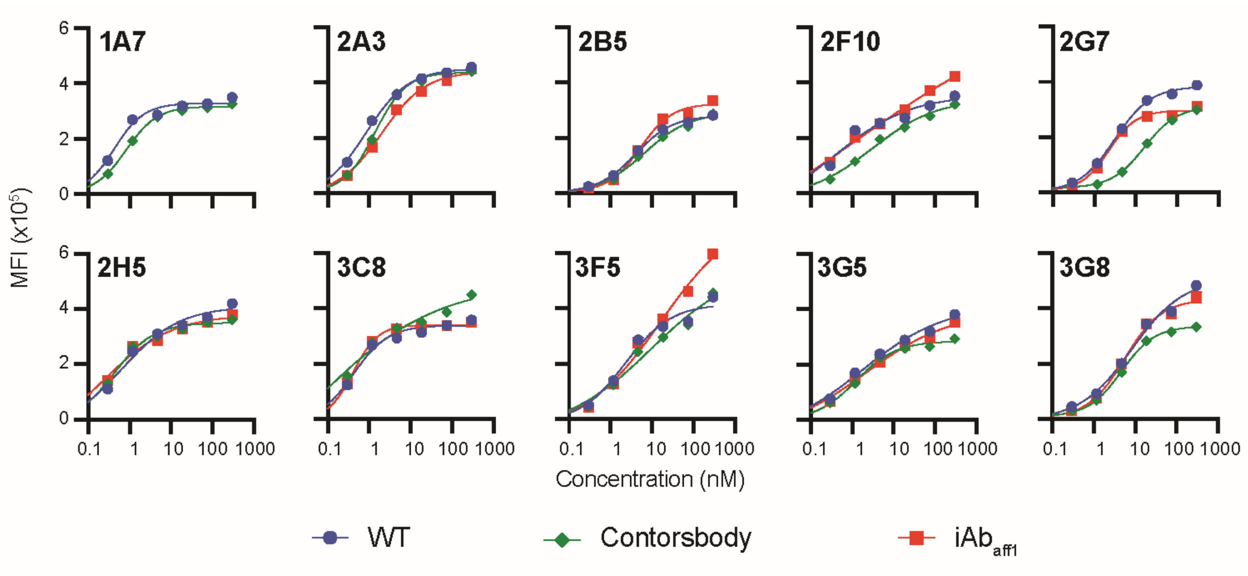

Supplementary Figure 6. Anti-OX40 cell binding titrations. Binding of each anti-OX40 clone as WT IgG, contorsbody and iAb$_{aff1}$ to OX40+ Jurkat cells was detected by FACS with a fluorescently labeled anti-human IgG Fab (n=2 independent wells). Source data are provided as a Source Data file.

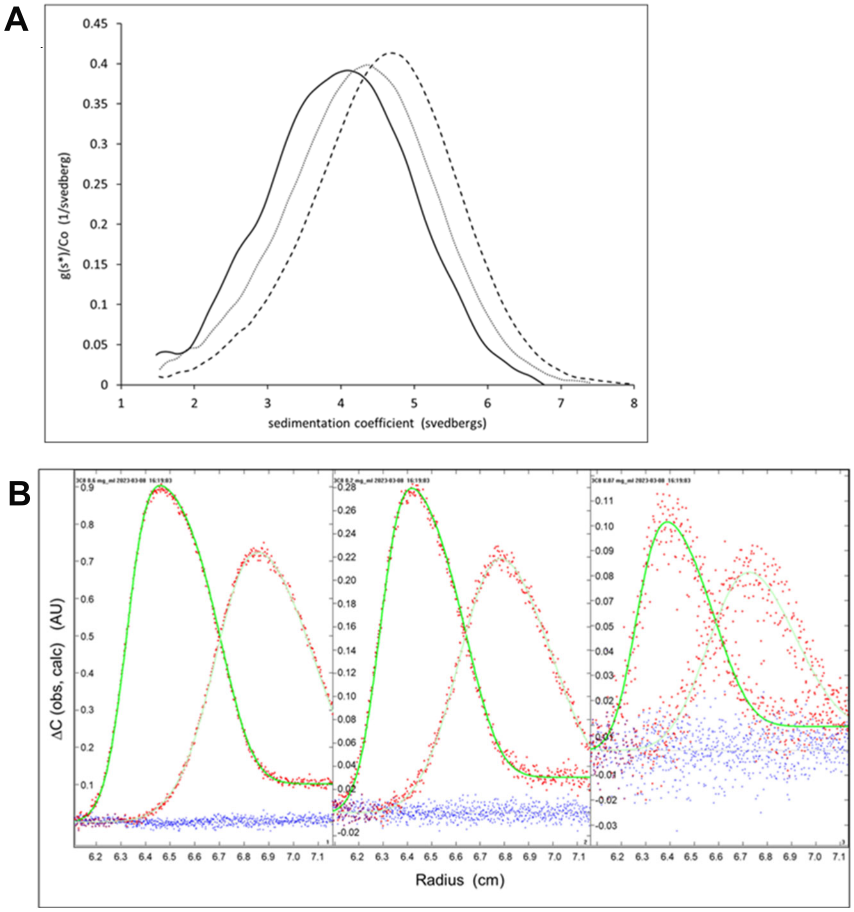

Supplementary Figure 7. Solution conformation of 3C8 iAb_{aff1} Fab determined by analytical ultracentrifugation. (A) *g(s*)* distributions of 0.6 mg/ml (dashed trace), 0.2 mg/ml (dotted trace) and 0.07 mg/ml (solid trace) 3C8 iAb_{aff1} Fab over the same reduced sedimentation time for each concentration, normalized for loading concentration. (B) Global fit to a monomer – dimer equilibrium from the same three protein concentrations as in (A). Shown for each concentration are the first and last ordered sets out of the 42 sets used in the fit. The red points are concentration differences, *ΔC(obs)*, at constant radius between two absorbance scans taken at different times. The solid green lines are concentration differences, *ΔC(calc)*, calculated from the parameters being fit. The *ΔC(obs)* and *ΔC(calc)* correspond to the left y-axis in units of absorbance at 280 nm. The plotted deviations between the observed and calculated concentration differences (blue points) are on the right y-axis using the same units and scale but offset to be at the bottom of each plot. The x-axis, radius of the cell in cm, is the same for both y-axes.

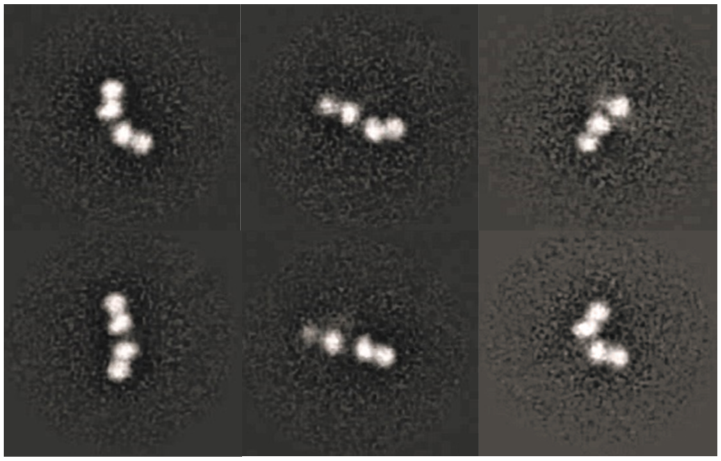

Supplementary Figure 8. Negative stain electron microscopy 2D classification images of the anti-OX40 antibody 3C8 with the iAb_{aff1} residue set in the $F(ab')_2$ format. While the full-length IgG format of 3C8 iAb_{aff1} adopts the i-shaped conformation (Fig. 2A), none of the 2D classes of the $F(ab')_2$ show intramolecular Fab-Fab association. The representative images shown are from a single experiment and data collection.

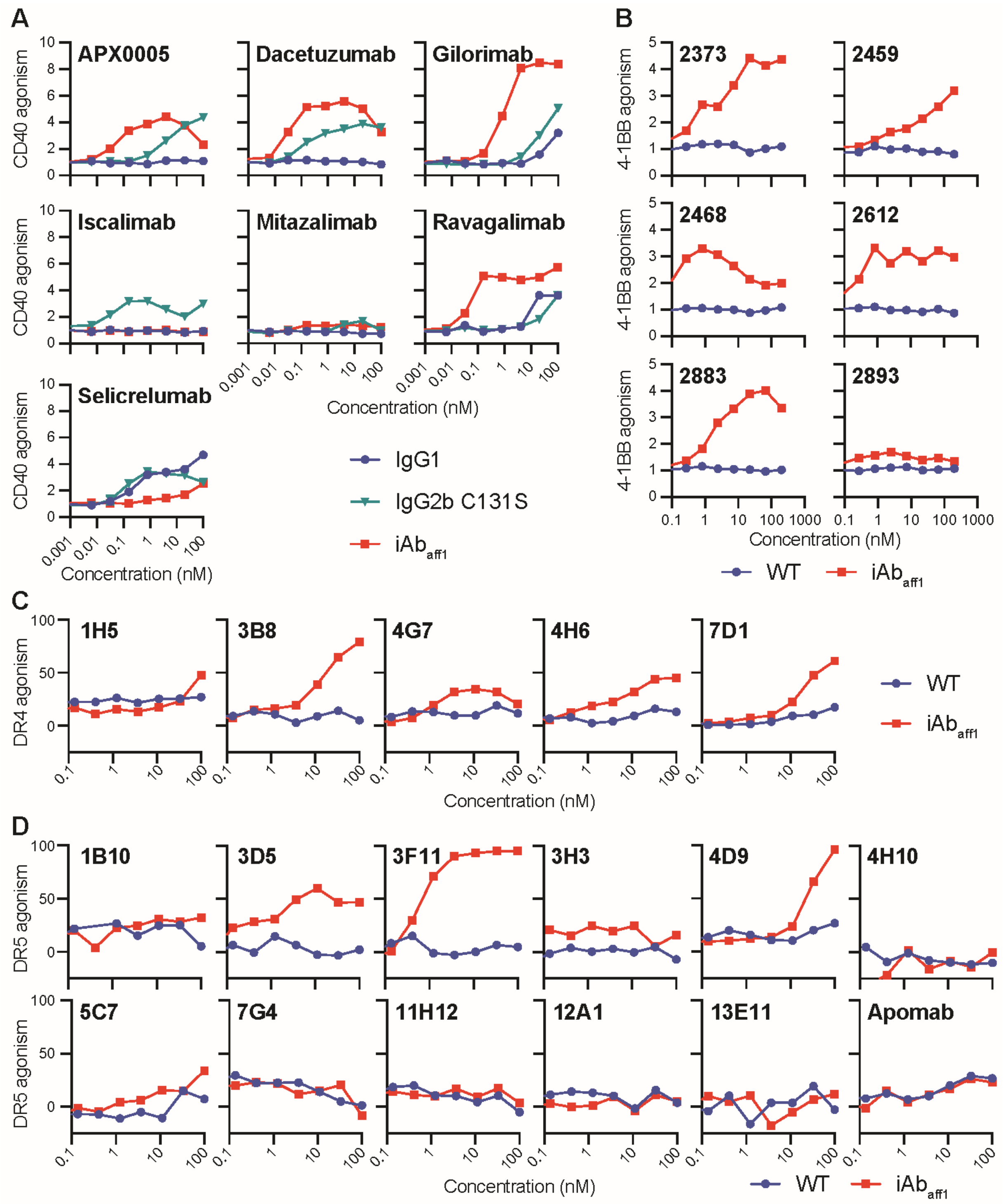

Supplementary Figure 9. iAb-induced agonism across 4 TNFRSF members. Individual activity titrations are shown of various formats for antibody clones against CD40 (A), 4-1BB (B), DR4 (C), and DR5 (D). CD40 and 4-1BB agonism are shown as fold change over an untreated control, while DR4 and DR5 agonism are shown as % killing relative to an untreated control (n=2 independent wells). Each anti-CD40 clone was produced as a WT human IgG1, human IgG2 C131S, and iAb_{aff1}, while all clones against other targets were only produced as a WT IgG1 and iAb_{aff1}. Source data are provided as a Source Data file.

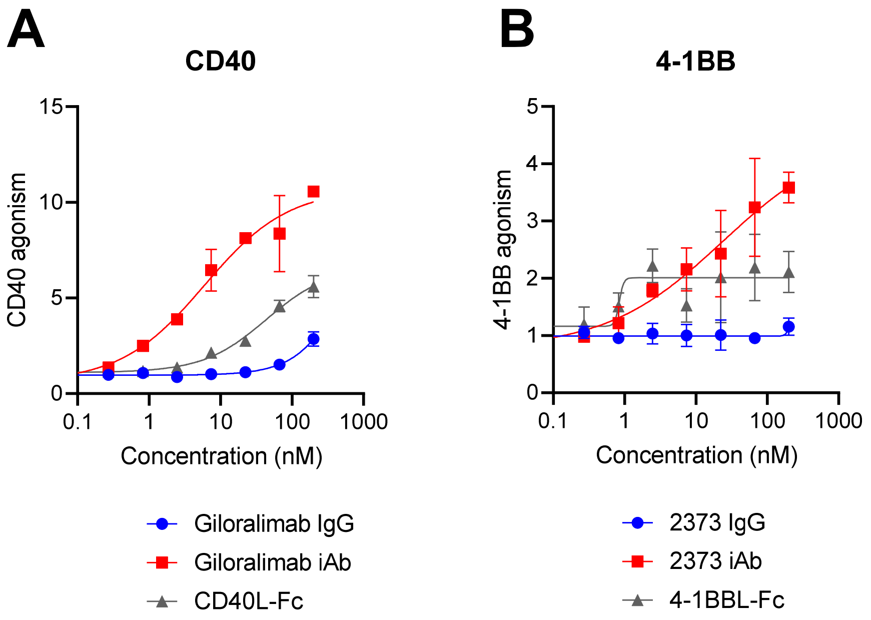

Supplementary Figure 10. Agonist activity of additional native TNFRSF ligands versus iAb_{aff1} format. Comparison of agonist activity for the native ligands CD40 (A) or 4-1BB (B) with WT IgG or iAb_{aff1} formats of an antibody clone for each TNFRSF member. The data are shown as fold change over an untreated control (n=3 independent wells) and presented as mean values +/- SEM. Source data are provided as a Source Data file.

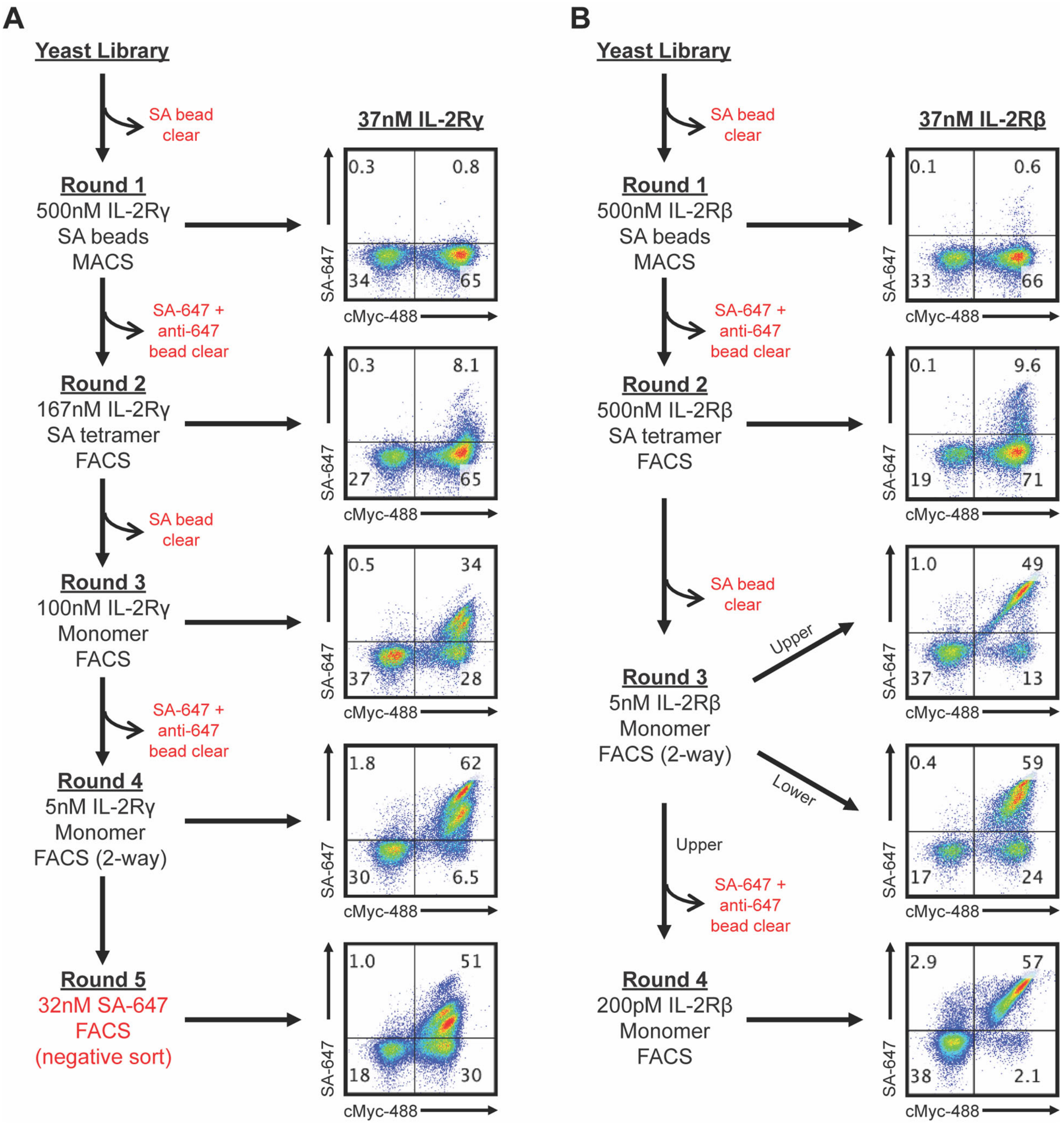

Supplementary Figure 11. Yeast selection overview. The schematics depict selection of IL-2Rβ (A) and IL-2Rγ (B) binders from an in-house derived scFv library displayed on yeast. For each antigen, selection was either magnetic- or FACS-based under increasingly stringent conditions with regard to both valency and concentration, as indicated. Red font shows rounds of negative selection. FACS plots at 37nM of each antigen show enrichment of binders after each round of selection. Axes show fluorescence signal from SA-647 and cMyc-488, representing antigen binding and surface scFv levels, respectively.

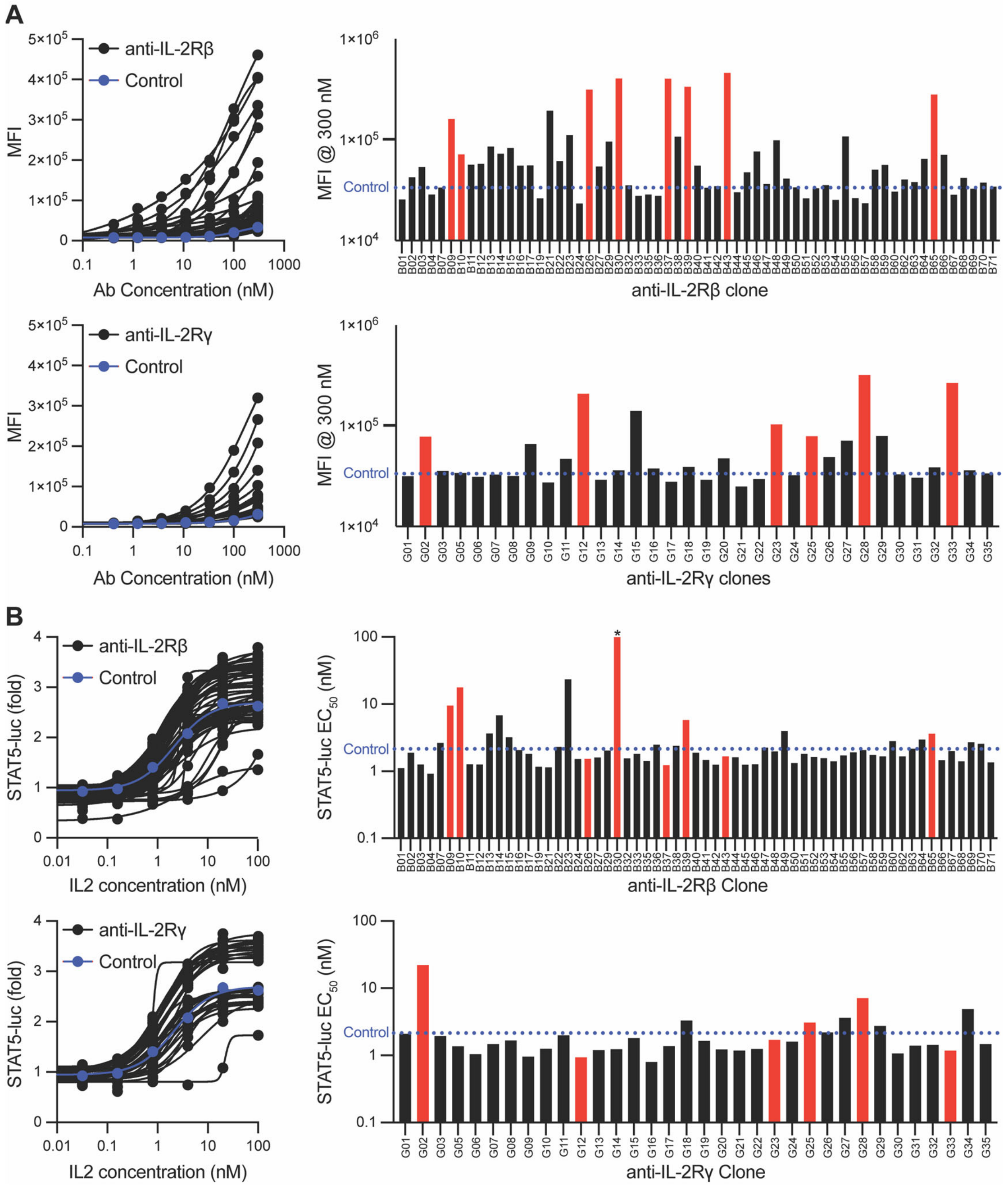

Supplementary Figure 12. Characterization of anti-IL-2Rβ and anti-IL-2Rγ antibody clones discovered by yeast display. Cell binding (A) and IL-2 blocking (B) analysis using Jurkat$^{\beta\gamma\text{-STAT5-Luc}}$ cells for anti-IL-2Rβ (top) and anti-IL-2Rγ (bottom) clones discovered by yeast display (n=1 sample per antibody). For IL-2 blocking experiments, cells were first coated with 1 µM of each monospecific anti-IL-2Rβ or anti-IL-2Rγ clone for 1 hour prior to the addition of an IL-2 serial dilution. For each analysis the anti-HER2 antibody, trastuzumab, was used as a negative control and shown in blue. The bar graphs on the right of each dose titration present the MFI at 300 nM and IL-2 EC_{50} of each antibody clone. Lead clones selected for bispecific reformatting are shown in red. The asterisk in the bottom graph denotes an EC_{50} greater than 100 nM. Source data are provided as a Source Data file.

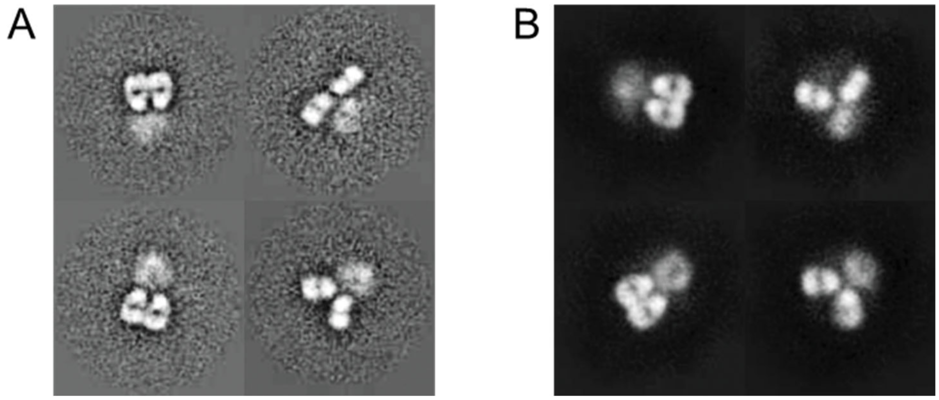

Supplementary Figure 13. Analysis of i-shaped antibody formation for two representative bispecific anti-IL-2R antibodies. Negative stain electron microscopy 2D classification images of the B10/G28 (A) and the B09/G28 (B) bispecific antibodies with the iAb_{aff1} residue set. The former demonstrated IL-2 agonist activity, while the latter did not (Fig. 5A). For the B10/G28 and B09/G28 iAb_{aff1} bispecific antibodies, 39% and 59% of the particles, respectively, adopt the i-shaped conformation, while the remaining particles are Y-shaped. The representative images shown are from a single sample preparation and data collection per antibody.

Supplementary Table 1. Parameters calculated from monomer – dimer equilibrium fit to AUC data

Parameter	Value
Monomer molecular weight	47.2 kDa
Monomer sedimentation coefficient	3.5 s
Dimer sedimentation coefficient	4.9 s
Equilibrium dissociation constant	6.8 μM
Standard deviation of the fit	0.011 AU

Supplementary Table 2. Surface plasmon resonance analysis of anti-IL-2Rβ and anti-IL-2Rγ clones

Clone	KD (nM)	Rmax
B01	NB	NB
B02	31.044	39.7
B03	53.915	11.8
B04	NB	NB
B07	NB	NB
B09	10.515	37
B10	2.866	106.1
B11	NB	NB
B12	NB	NB
B13	5.939	93.8
B14	5.479	78.8
B15	26.812	50.1
B16	20.245	67.6
B17	18.455	38.3
B19	NB	NB
B21	NB	NB
B22	22.983	63.1
B23	NB	NB
B24	NB	NB
B26	6.538	68
B27	23.019	63.1
B29	20.953	14.6
B30	13.977	41.5
B32	26.008	56.3
B33	73.597	7.4
B35	NB	NB
B36	NB	NB
B37	36.143	7.2
B38	NB	NB
B39	3.995	70.7
B40	NB	NB
B41	NB	NB
B42	NB	NB
B43	13.255	41.6

Clone	KD(nM)	Rmax
B44	NB	NB
B45	NB	NB
B46	NB	NB
B47	NB	NB
B48	26.434	11.1
B49	17.921	54.8
B50	NB	NB
B51	NB	NB
B52	NB	NB
B53	NB	NB
B54	NB	NB
B55	26.768	12.5
B56	NB	NB
B57	NB	NB
B58	NB	NB
B59	8.762	34
B60	NB	NB
B62	NB	NB
B63	NB	NB
B64	39.233	10.7
B65	45.233	11.7
B66	NB	NB
B67	NB	NB
B68	NB	NB
B69	33.321	46.3
B70	NB	NB
B71	63.981	11.9

Clone	KD (nM)	Rmax
G01	NB	NB
G02	9.595	71.5
G03	37.585	38
G05	NB	NB
G06	NB	NB
G07	NB	NB
G08	32.351	17.4
G09	NB	NB
G10	NB	NB
G11	14.053	4.8
G12	52.694	2.3
G13	NB	NB
G14	48.288	12.8
G15	NB	NB
G16	NB	NB
G17	36.651	20.8
G18	128.12	5.8
G19	25.488	9.5
G20	NB	NB
G21	36.436	7.7
G22	NB	NB
G23	41.21	33.2
G24	NB	NB
G25	47.436	13.4
G26	6.935	10.9
G27	18.451	6.6
G28	68.679	14.1
G29	39.414	6.3
G30	NB	NB
G31	NB	NB
G32	NB	NB
G33	19.852	16.5
G34	131.23	63.2
G35	37.234	4.7
